# Description of a new troglomorphic species of *Charinus* Simon, 1892 from Brazil (Arachnida, Amblypygi, Charinidae)

**DOI:** 10.3897/zookeys.600.8580

**Published:** 2016-06-22

**Authors:** Ana Caroline Oliveira Vasconcelos, Alessandro Ponce de Leão Giupponi, Rodrigo Lopes Ferreira

**Affiliations:** 1 Centro de Estudos em Biologia Subterrânea, Departamento de Biologia, Universidade Federal de Lavras, Lavras-MG, CEP 37200-000, Brazil; 2 Laboratório de Referência Nacional em Vetores das Riquetsioses (LIRN), IOC-FIOCRUZ, Manguinhos, Rio de Janeiro-RJ, CEP 21040-360, Brazil

**Keywords:** Neotropics, subterranean species, taxonomy, whip spider

## Abstract

*Charinus taboa* **sp. n.** comprises the twenty-second species of the genus described for Brazil. The new species belongs to the eastern Brazilian group, in which all species have sucker-like gonopods. *Charinus taboa* **sp. n.** has a marked sexual dimorphism in the pedipalps as do other members of the genus in the country. The description of *Charinus taboa* **sp. n.** offers an opportunity to discuss some aspects of ecology, troglomorphism and conservation within the genus. A key to the eastern Brazilian species of *Charinus* is provided.

## Introduction

Knowledge of the Amblypygi fauna remained low and fairly constant for many years until last decade of the 20^th^ century, when studies and descriptions of whip spiders considerably increased in number (Harvey 2007). Yet, until 2015, the number of known species of *Charinus* in Brazil was just 11, but it has rapidly almost doubled over the last year, reaching 21 described species (Vasconcelos and Ferreira 2016, Giupponi and Miranda 2016). The most specious and widely distributed group of Amblypygi is the genus *Charinus*. It is found throughout the tropics, including diverse types of habitat and even oceanic islands (Weygoldt 2000, Miranda and Giupponi 2011, Jocque and Giupponi 2012, Harvey 2013, Vasconcelos et al. 2013, 2014, Réveillion and Maquart 2015). However, the considerable richness of the genus (more than 60 species) allied with its wide distribution and distinct morphologies of the female genitalia suggest that the genus is not monophyletic (Weygoldt 1996, 2000, Harvey 2013).

Species of the genus *Charinus* are dependent on moist environments, and as other whip spiders, they are of nocturnal habit (Weygoldt 2000, Vasconcelos et al. 2013). Together with their flattened bodies, one of the characteristics of Amblypygi, *Charinus* species also have small body sizes (up to 16mm), which allow them to shelter in a range of microhabitats, such as trunks, bromeliads, rocks, subterranean cavities and termite nests (Weygoldt 2000, Jocque and Giupponi 2012, Vasconcelos et al. 2014, Réveillion and Maquart 2015). Synanthropic habitats can also house some species of *Charinus*, such as *Charinus vulgaris* Miranda and Giupponi, 2011, and the parthenogenetic species, *Charinus acosta* (Quintero, 1983) and *Charinus ioanniticus* (Kristcher, 1959) (Armas 2005, Weygoldt 2005).

In Brazil, *Charinus* species typically use caves as habitat. Within the 21 known species of the genus in the country, 13 were described from individuals collected in caves: *Charinus acaraje* Pinto-da-Rocha, Machado and Weygoldt, 2002, *Charinus mysticus* Giupponi and Kury, 2002, *Charinus troglobius* Baptista and Giupponi, 2002, *Charinus eleonorae* Baptista and Giupponi, 2003, *Charinus potiguar* Vasconcelos, Giupponi and Ferreira, 2013, *Charinus jibaossu* Vasconcelos, Giupponi and Ferreira, 2014, *Charinus caatingae* Vasconcelos and Ferreira, 2016, *Charinus iuiu* Vasconcelos and Ferreira, 2016, *Charinus ricardoi* Giupponi and Miranda 2016, *Charinus carajas* Giupponi and Miranda 2016, *Charinus orientalis* Giupponi and Miranda 2016, *Charinus ferreus* Giupponi and Miranda 2016 and *Charinus bichuetteae* Giupponi and Miranda 2016. Among these species, *Charinus troglobius*, *Charinus eleonorae*, *Charinus caatingae* and *Charinus ferreus* represent hitherto the only strictly cavernicolous species in the country. Herein, a new species of *Charinus* is described from limestone caves located in Sete Lagoas, state of Minas Gerais, Brazil. Moreover, the possible troglobitic status of this species is discussed.

## Methods

The specimens were collected through visual searches throughout floors and walls of the caves. All specimens were captured with a fine forceps and placed in vials containing 70% ethanol.

The description of the species was based on the entire type material. Measurements and general terminology were based on the proposals of Quintero (1981). The terminology of pedipalps and legs followed Harvey and West (1998). The tarsus as defined by Harvey and West (1998) is divided here into tarsus and claw (apotele), as there is no fusion of these two segments in Charinidae. Measurements of the pedipalps were taken between the condyles of each segment. Measurements were taken of the entire type-series (quantity indicated as “n”), presenting first their mean values followed by the range of variation in parentheses. The terminology of the structures of male gonopods followed Giupponi and Kury (2013).

The following abbreviations are used:

BT
 basitibia 


DT
 distitibia 


GO
 genital operculum 


Fi
 fistula (gonopod tube) 


Pi
 processus internus 


LaM
 lamina medialis 


LoD
 lobus dorsalis 


LoL1
 lobus lateralis primus 


LoL2
 lobus lateralis secundus 


Photographs were taken using a Leica M205A stereomicroscope with the software Leica Application Suite Automontage. Illustrations of the male and female gonopods were made using a camera lucida coupled to a Leica MDLS phase contrast microscope.

The specimens were deposited in the following institution collections:

MNRJ
 Museu Nacional, Rio de Janeiro, Brazil 


ISLA
 Seção de Invertebrados Subterrâneos, Coleção de Zoologia of the Universidade Federal de Lavras, Minas Gerais, Brazil 


CAVAISC
Fundação Oswaldo Cruz, Instituto Oswaldo Cruz, Rio de Janeiro, Brazil 


DNS
 Geographical coordinates are given in Degrees, Minutes and Seconds 


## Taxonomy

### Charinidae Quintero, 1986

#### Charinus

Taxon classificationAnimaliaAmblypygiCharinidae

Simon, 1892

##### Type-species.

*Phrynus australianus* L. Koch, 1867, by original designation.

#### Charinus taboa sp. n.

Taxon classificationAnimaliaAmblypygiCharinidae

http://zoobank.org/5F363344-B51E-4F55-872E-09AB75E8F2F3

Figs 1–3 , 4–8 , 9–13 , 14–17 , 18–19 , 20–23

##### Type-locality.

BRAZIL, Minas Gerais: Sete Lagoas, 19°28'29.68"S, 44°19'41.31"W, Taboa Cave and BR 24 cave (19°27'59.89"S 44°19'48.47"W)

##### Type-material.

Holotype male (MNRJ 09091) from Taboa cave (19°28'29.68"S, 44°19'41.31"W), Sete Lagoas, Minas Gerais, Brazil, 15 Sept. 2005, R. L. Ferreira leg. Paratypes: juvenile female (MNRJ 09092), juvenile female (MNRJ 09092), female (ISLA 4019), female (ISLA 4020), female (ISLA 4021), male (ISLA 4022), male (ISLA 4023), juvenile male (ISLA 4024), juvenile male (ISLA 4030) with the same data as holotype, female and male (CAVAISC-ARAC 0007) from BR 24 cave (19°27'59.89"S, 44°19'48.47"W), Sete Lagoas, Minas Gerais Brazil, 22 Jun. 2015, F. Bondezan leg. and female (CAVAISC-ARAC 0008) from BR 24 cave, Sete Lagoas, Minas Gerais Brazil, 18 Dec. 2015, F. Bondezan leg.

##### Diagnosis.

*Charinus taboa* differs from other species of the genus by the following combination of characteristics: frontal process with thickened apex; median eyes reduced, with flattened tubercle; lateral eyes not developed and without pigmentation (little pigmentation in smaller individuals); tritosternum with a slightly forked apex; pedipalps sexually dimorphic; femur of the pedipalp with 4-5 dorsal spines (typically 5) and 5-6 ventral spines (typically 5); patella of the pedipalp with 6-7 dorsal spines (typically 6) and 4 ventral spines; distitibia of the leg IV with 16 trichobothria; female gonopod sucker-like, with irregular opening and edges with a small fold; male gonopod with pairs of Pi and LoL1 emerging from each side of the Fi with thin prolongations, and pairs of LoD and LoL2 claw-shaped emerging from the interior of the upper portion of Fi.

##### Description.

**Figures 1–3.**
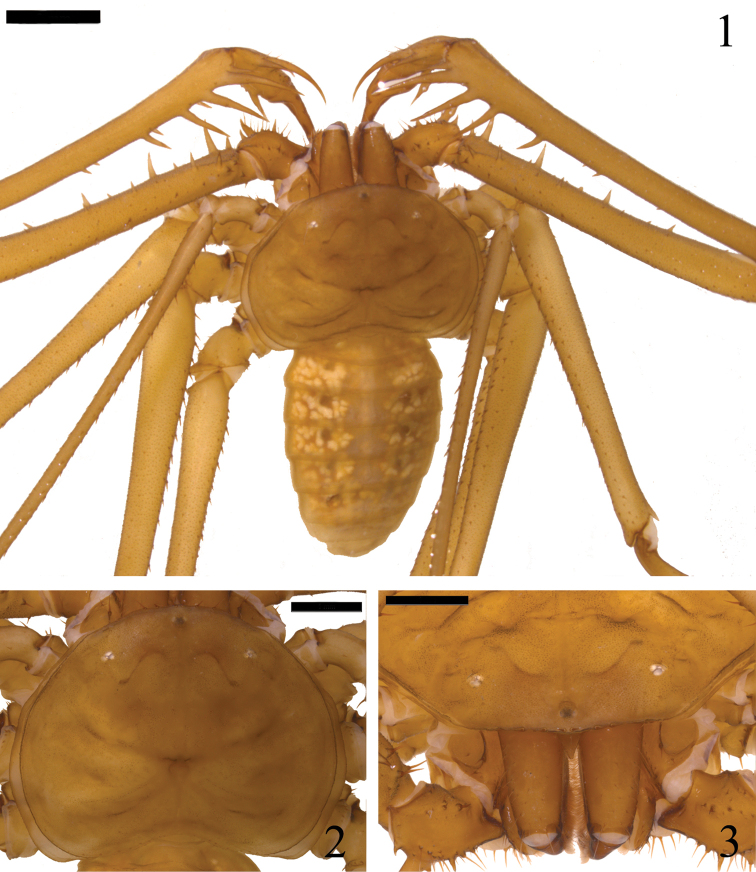
*Charinus taboa* sp. n. Holotype: **1** Habitus. **2** Carapace **3** Frontal view of the carapace and frontal process. Scale bars: **1** = 2 mm; **2, 3** = 1 mm.

*Carapace* (Figs 1–3, 9): Flattened. Wider than long (ratio length/width a little less than 3/4). Anterior margin rounded with corners flattened downwards. Six strong setae on the anterior margin projected upwards (one extra seta is found centrally in a female), the central two setae usually located directly in front of the tubercle of the median eyes. Frontal process triangular in shape, with thickened apex and visible in dorsal view. Carina begins at the corners of the anterior margin and extends from the coxae of leg II to the corners of the posterior margin. Median eyes reduced, with flat tubercle. Lateral eyes not very developed, without pigmentation (less pigmentation in smaller specimens) and with one seta posterior to each triad. Frontal hump present at each side, starting just at the front of the lateral eyes and ending in a depression located at each side of the carapace. Fovea located posterior to the center, from which radiate two pairs of furrows in anterior and posterior orientation like an “X”. Median depression located on each side between these two pairs of furrows. A thin furrow follows medially from the median eye tubercle and reaches the posterior margin. Punctuations arranged in lines and spots, more densely in the anterior region.

*Sternum* (Figs 8, 11): Tri-segmented with all segments sclerotized and convex. Tritosternum projected anteriorly, elongated, cone-shaped, with slightly forked apex, with one apical pair of strong setae and three median strong setae in the holotype, and one apical, one medial and one basal pair of strong setae in the paratypes. Few setae along the tritoesternum. Second segment (mesosternum) rounded, with one strong seta at each upper corner and few setulae encircling the base. Third segment (metasternum) rounded, with one strong seta at each upper corner and few setulae encircling the base. The segments are separated from each other approximately by the diameter of the mesosternum.

**Figures 4–8.**
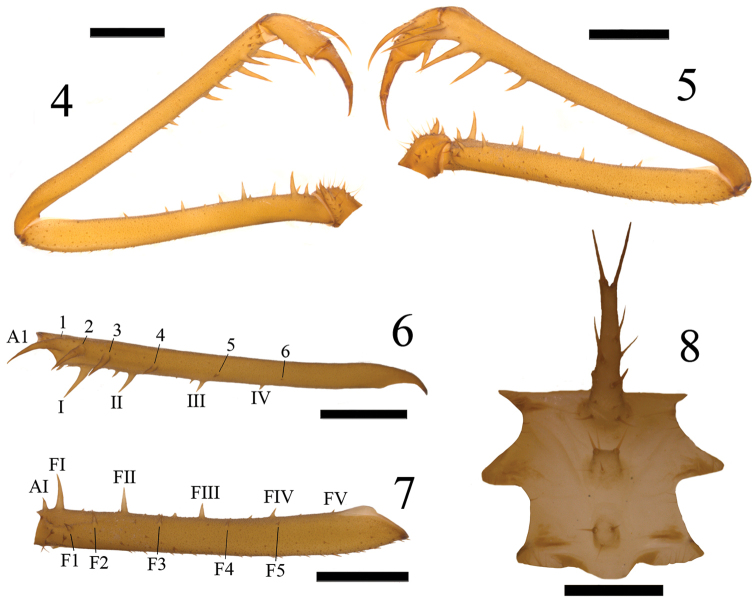
*Charinus taboa* sp. n. Holotype: **4** Right pedipalp in ventral view **5** Right pedipalp in dorsal view **6** Patella of the pedipalp in dorsal view with spines indicated **7** Femur of the pedipalp in dorsal view with spines indicated **8** Sternum. Scale bars: **4–7** = 2 mm; **8** = 500 µm.

**Figures 9–13.**
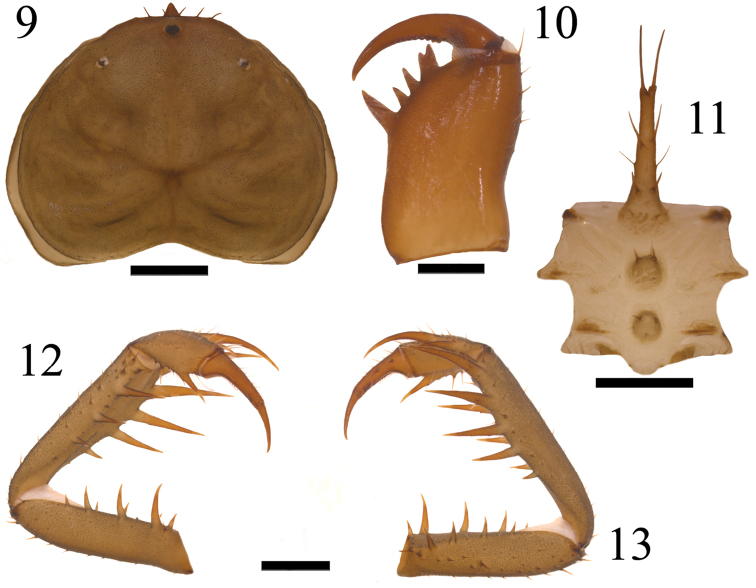
*Charinus taboa* sp. n. Female paratype: **9** Carapace **10** Right chelicerae **11** Sternum **12** Right pedipalp in ventral view **13** Right pedipalp in dorsal view. Scale bars: **9, 12, 13** = 1 mm; **10, 11** = 500 µm.

*Abdomen* (Fig. 1): Oblong, with punctuations barely distinguishable.

*Chelicera* (Fig. 10): Cheliceral furrow with 4 inner teeth. The distal tooth is bifid, the distal cusp being larger than the proximal. Teeth length: IV>Ia>Ib=II>III. Claw with 8-9 denticles. Dorsal setae located distally and in the inner side of the chelicerae.

*Pedipalp* (Figs 4–7, 12, 13): Trochanter: ventral spiniform apophysis pointed forwards with a series of subequal setiferous tubercles; two spines of subequal length located aligned on the prolateral face, the first being near the medial region and the second dorsally to the projection of the apophysis and close to the femur; three setae aligned between the spines and two located basally to the first spine; dorsal oblique series of strong setae. Femur: dorsal portion with three strong setiferous tubercles on the basal region, one being located more ventrally; several strong setae along the segment; four-five dorsal spines (typically five) decreasing in size: F1>F2>F3>F4>F5; five-six ventral spines (typically five) of sizes: FI>FII>FIII>AI>FIV>FV; some secondary spines are present in males between the spines on the dorsal and ventral sides. Patella: some strong dorsal setae between the spines; six-seven dorsal spines (typically six) of sizes: 1>2>3>4>A1>5>6; large ventral setiferous tubercles located distally: four ventral spines of sizes: I>II>III>IV; some secondary spines between the ventral spines in males. Tibia: strong dorsal setae; two dorsal spines, the second being approximately two times larger than the first; strong ventral setae on the basal portion; one ventral spine located on the distal half of size slightly smaller than the dorsal spine one. Tarsus: strong dorsal setae and some long ventral setae; two dorsal spines on the cleaning organ, the second being approximately two times larger than the first spine. Cleaning organ occupies about half the length of the article. Claw (apotele): long with sharp curved tip.

*Legs*: all densely setose. Femur lengths: I>III>II>IV. Leg I: tibia with 23 articles and tarsus with 41 articles. Leg IV: basitibia with four pseudo-articles and one trichobothrium located basally on the last article. Distitibia (Fig. 17) with three basal and 13 distal trichobothria; frontal and caudal series with five trichobothria each. Basitibia-distitibia length: BTI>DT>BT4>BT3>BT2. Ratio tarsus/metatarsus approximately 3/4. Tarsus tetramerous.

**Figures 14–17.**
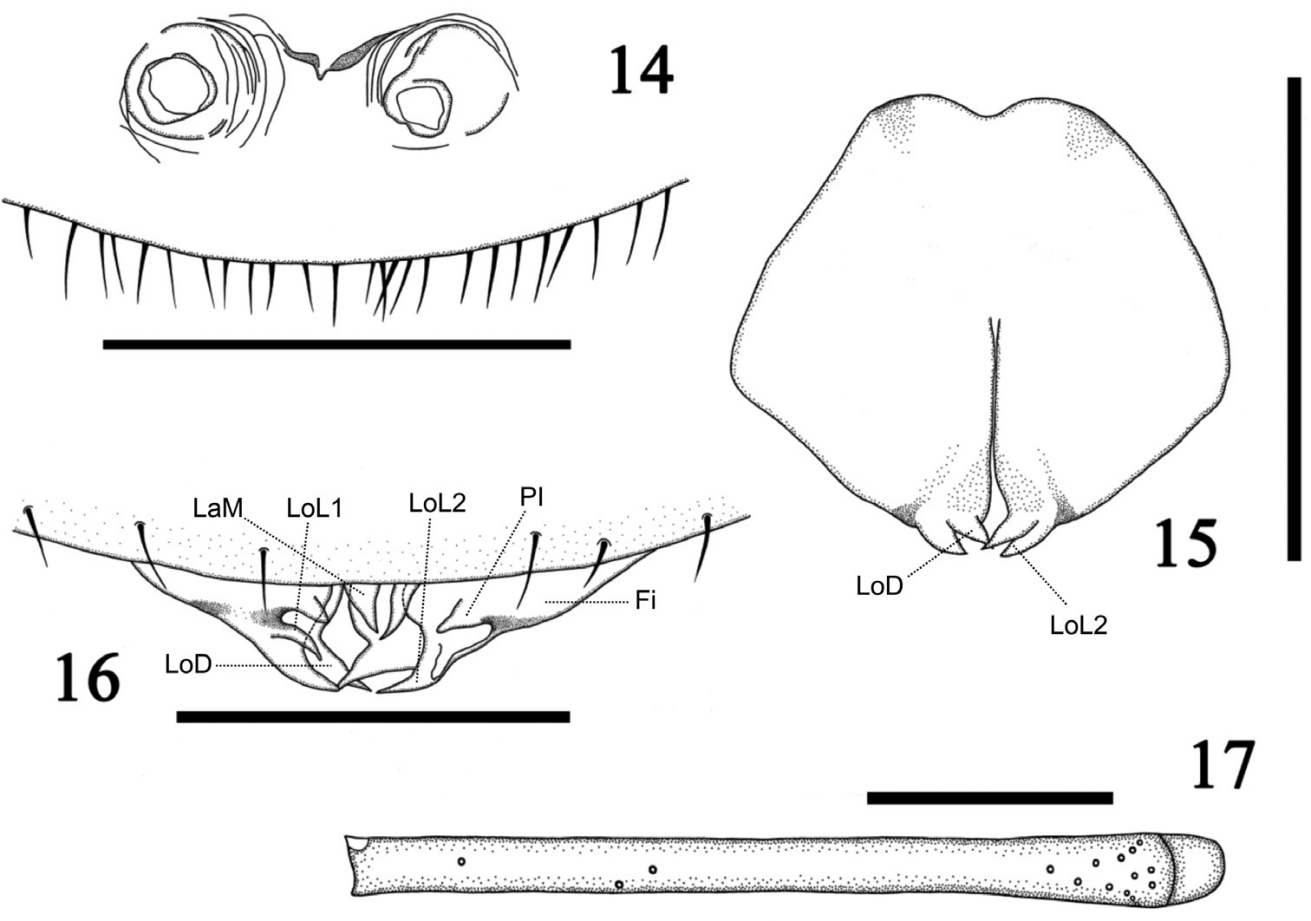
*Charinus taboa* sp. n. Female paratype: **14** Gonopod. Holotype: **15** Dorsal view of the gonopod **16** Ventral view of the gonopod with structures indicated: Fi = fistula (gonopod tube), Pi = processus internus, LaM = lamina medialis, LoD = lobus dorsalis, LoL1 = lobus lateralis primus, LoL2 = lobus lateralis secundus **17** Distitibia of the right leg IV. Scale bars: **14, 16** = 500 µm; **15, 17** = 1 mm.

**Figures 18–19.**
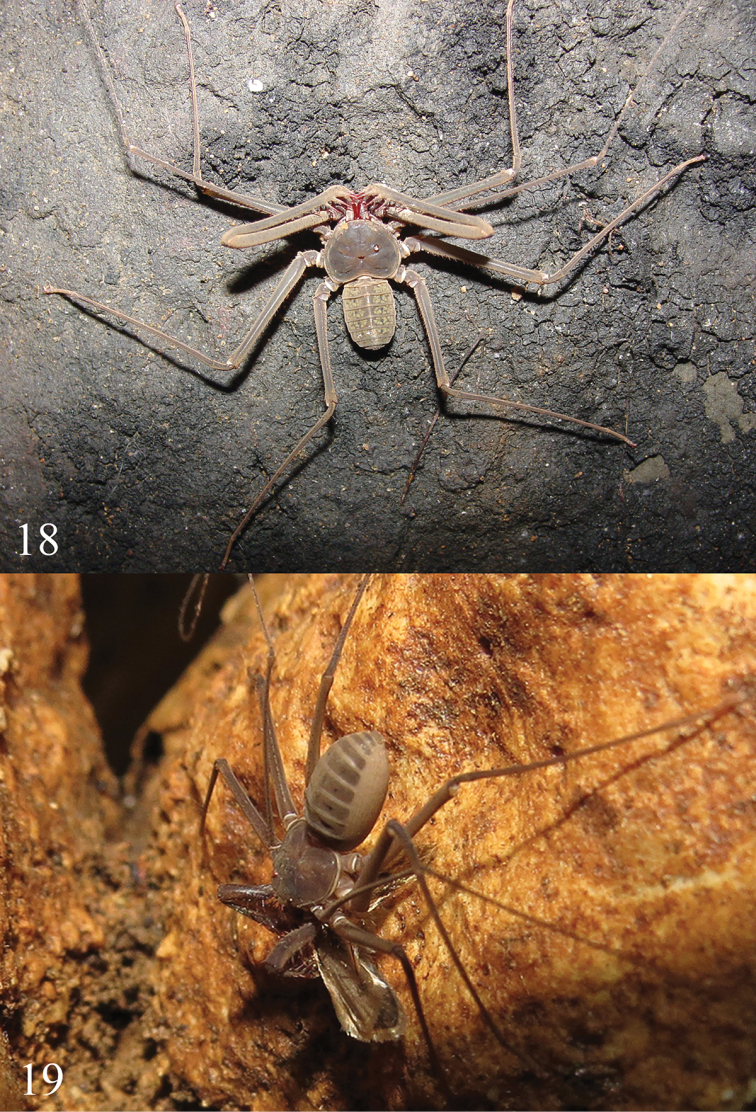
*Charinus taboa* sp. n. **18** Male inside the Taboa cave **19** Female preying a moth (Noctuidae).

*Color* in live specimens (Figs 18, 19): body light brown. In alcohol (Fig. 1): body yellowish; some specimens exhibit slightly reddish coloration on the carapace, pedipalps, chelicerae and legs.

*Male genitalia* (Figs 15, 16): GO rounded with few scattered setae. Genitalia a little wider than long. Longitudinal split occupies about half of the genital organ. Fi exceeds the genital operculum margin. Sclerotized band surrounds each side of the Fi, reaching the Pi and LoL1. Pair of Pi and LoL1 emerges in thin prolongations from each side of the Fi. Pair of LoD and LoL2 claw-shaped emerges from the interior of the upper portion of Fi, with LoL2 being ventral to the LoD. Pair of LaM claw-shaped and smaller, located ventrally to the Fi.

*Female genitalia* (Fig. 14): Rounded genital operculum margin with many strong setae. Gonopods sucker-like, barrel shaped and slightly wider than long. Irregular gonopods opening, with edges with a small fold retracted in a portion between the gonopods and the operculum margin, and with a bottleneck below the edges. Gonopods separated from each other by a distance smaller than the diameter of each one and from the margin of the operculum by a distance larger than its length.

##### Etymology.

The specific epithet is treated as a noun in apposition and refers to the name of the cave (Taboa) where most of the specimens were collected.

##### Distribution.

The new species is known from the Taboa and BR 24 caves, state of Minas Gerais, Brazil.

##### Ecology.

Amblypygids perform their vital activities, such as mating and feeding, in nocturnal periods. The most important sensory organ used by whip spiders for capturing prey is the antenniform leg, while the eyes are most important for avoiding light (Weygoldt 2000, Pinto-da-Rocha et al. 2002). This way, amblypygids can be considered pre-adapted to subterranean life, since they are able of searching for food in a completely darkness.

Eyes in Amblypygi are also important for adjusting to circadian rhythms (Weygoldt 2000). After many generations living in a subterranean habitat, some hypogean animals might have their activity period modified, as shown for some species of fish (Menna-Barreto and Trajano, 2015). This possible change in behavior may have occurred in *Charinus taboa*, as one specimen was observed preying on a Noctuidae (Lepidoptera) during the day (Fig. 19). Nevertheless, we cannot discard the possible scenario where the common ancestral of the clade where *Charinus taboa* belongs was a species that have diurnal activity of alimentation.

Contrarily to that observed, moths were considered by Weygoldt (2000) as typical preys of *Heterophrynus* Pocock, 1894, which are agile “sit and wait” predators, as opposed to animals of small body size, as *Charinus*, which behave as active predators. In ground habitats, amblypygids also act as one of the largest predators in these environments, as the fauna of underground cavities consists mostly of small arthropods (Culver and Pipan 2009). Other invertebrates, as crickets and cockroaches, have been cited as potential preys of *Charinus* in caves (Vasconcelos et al. 2013, Vasconcelos and Ferreira 2016).

**Figure 20–23.**
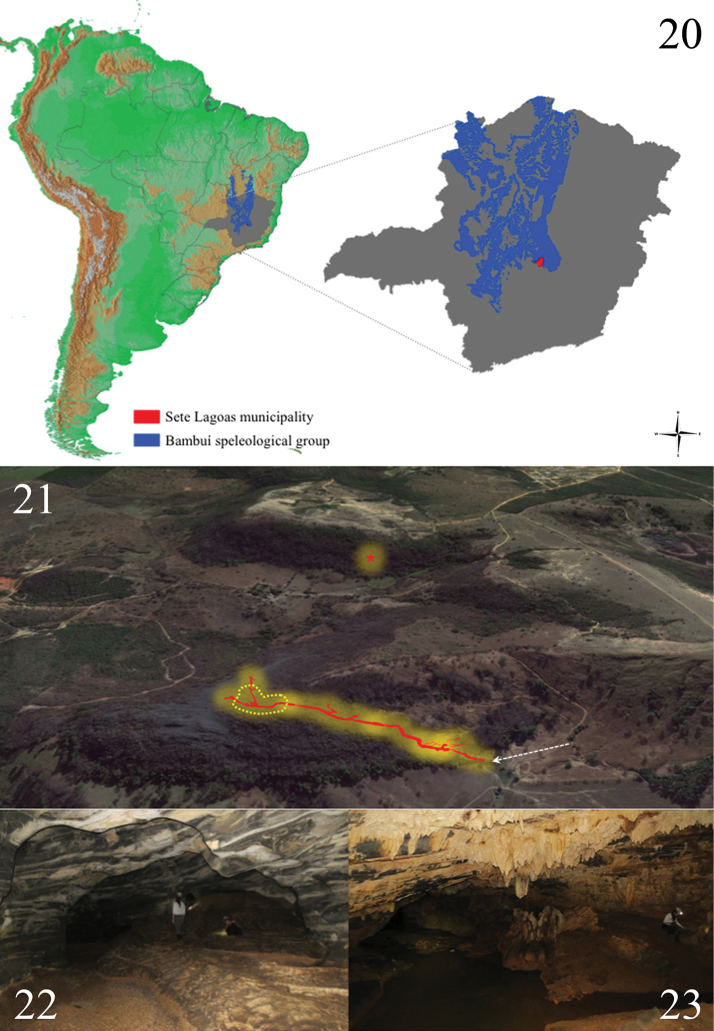
**20** Locality of Sete Lagoas (municipality where are located the Taboa and BR 24 caves) in the state of Minas Gerais, Brazil. The blue area corresponds to the Bambui limestone group and the red area correspond to the Sete Lagoas municipality **21** Location of the Taboa cave (the arrow indicates the main entrance of Taboa cave and the circle the location where individuals of *Charinus taboa* were found) and BR-24 cave (star represents the entrance) **22** Portion of the Taboa cave where specimens were collected **23** Portion of the Taboa cave with a watercourse where most of the specimens were found.

Specimens of *Charinus taboa* were only found in two caves (Taboa cave and BR 24 cave), both located in the Bambui speleological group and near the city of Sete Lagoas (Fig. 20), in a zone with pronounced anthropization (Fig. 21). The external native vegetation was quite modified, with fragments of vegetation associated only with rocky outcrops, which comprises inappropriate areas for agriculture. Nevertheless, many outcrops were altered anthropically or completely destroyed by mining activities. Tens of caves were target of biospeleological inventories in the region where Taboa and BR 24 caves are located (R. Ferreira, data not published). Nonetheless, individuals of *Charinus taboa* were not found in other localities than the cited caves. Such caves are quite close to each other, with the distance between them less than 500 meters (both caves are associated to a continuous limestone outcrop) (Fig. 20). It is important to point that although both caves (Taboa and BR-24) are not connected by macro-spaces, it is plausible to assume the existence of meso-caverns in between them. Such small spaces would certainly allow the movements of individuals through the underground between those caves.

The BR-24 cave is a small cave (33,8 meters long), with a single entrance and an isolated chamber in its deepest portion, where the specimens were found. This chamber is quite moist, even during the dry season. In total, 6 specimens were found in the dry season and only one specimen was observed in the rainy season. Specimens of *Charinus taboa* were observed in the cave walls and ceiling, always in the deepest portion of the cave. Potential preys include moths and crickets.

During the visit to the Taboa cave (which has around 800 meters long), about 15 adults and 10 juveniles were observed. The adults were mainly found between speleothems on walls and ceiling of the cave, while juveniles were seem frequently under rocks. This behavior of sheltering among speleothems and under rocks may eventually means a response to pressure of cannibalism or predation, since others predators of bigger size (as spiders of the genus *Isoctenus* Bertkau, 1880) cohabit the cave. This type of behavior was also registered in *Charinus potiguar* and in juveniles of *Heterophrynus cheiracanthus* (Gervais, 1844) in the night (Ladle and Velander 2003). All individuals of *Charinus taboa* were found in the inner portion of the cave, near to a large watercourse (Figs 22, 23). Similar preference was also observed in *Charinus troglobius* and *Charinus eleonorae* (Baptista and Giupponi 2002, 2003).

#### Key to eastern Brazilian species of *Charinus* (modified from Miranda and Giupponi 2011)

**Table:** 

1	Median eyes absent	***Charinus troglobius***
–	Median eyes present	**2**
2	Second and third sternal sclerites flattened and twice as wide as long (Espírito Santo: Domingos Martins)	***Charinus montanus***
–	Second and third sternal sclerites convex and roundish	**3**
3	Distitibia of leg IV with 18 trichobothria	**4**
–	Distitibia of leg IV with 16 trichobothria	**11**
4	Patella of the pedipalp with 2 ventral spines	**5**
–	Patella of the pedipalp with 3 or more ventral spines	**6**
5	Lateral eyes triads with pigmentation (Bahia: Santa Luzia, Gruta Pedra do Sino Cave)	***Charinus acaraje***
–	Lateral eyes triads without pigmentation (Rio Grande do Norte: Felipe Guerra, Buraco Redondo Cave)	***Charinus potiguar***
6	Median eyes tubercle indistinct (Minas Gerais: Itacarambi, Olhos d’Água Cave)	***Charinus eleonorae***
–	Median eyes tubercle distinct	**7**
7	Lateral eyes underdeveloped (Bahia: Várzea Nova: Fazenda Jurema Cave)	***Charinus caatingae***
–	Lateral eyes developed	**8**
8	Patella of the pedipalp with 3 ventral spines	**9**
–	Patella of the pedipalp with 4 or 5 ventral spines	**10**
9	Femur of the pedipalp with 3 or 4 dorsal spines (Bahia: Iuiu: Lapa do Baixão Cave)	***Charinus iuiu***
–	Femur of the pedipalp with 5 or 6 dorsal spines (Minas Gerais: Arcos: Gruta da Cazanga)	***Charinus jibaossu***
10	Tarsus of the pedipalp with 3 dorsal spines (Bahia: Gentio do Ouro, Encantados Cave)	***Charinus mysticus***
–	Tarsus of the pedipalp with 2 dorsal spines (São Paulo: Ilha Bela)	***Charinus asturius***
11	Lateral and median eyes developed with high tubercle (Espírito Santo: Serra)	***Charinus brasilianus***
–	Lateral and median eyes underdeveloped with low tubercle (Minas Gerais: Sete Lagoas: Taboa Cave)	***Charinus taboa* sp. n.**

## Discussion

As proposed by Weygoldt (2005, 2006, 2008), species of *Charinus* can be divided into four groups based on the morphology of the female gonopods, including species with “sucker-like” gonopods (*Charinus brasilianus* group), “cushion-like” gonopods (*Charinus australianus* group), a group of species with “finger-like” gonopods (*Charinus bengalensis* group), and finally, the group represented by *Charinus seychellarum*, in which the gonopods were totally lost. In Brazil, the species distributed on the eastern side are included in the *Charinus brasilianus* group, while species distributed in the Amazon region are considered part of the *Charinus australianus* group. *Charinus taboa* is placed in the first group in conjunction with *Charinus brasilianus* Weygoldt, 1972, *Charinus montanus* Weygoldt, 1972, *Charinus asturius* Pinto-da-Rocha, Machado & Weygoldt, 2002, *Charinus acaraje*, *Charinus mysticus*, *Charinus troglobius*, *Charinus eleonorae*, *Charinus potiguar*, *Charinus jibaossu*, *Charinus caantingae* and *Charinus iuiu. Charinus schirchii* (Mello-Leitão, 1931) is also located in eastern Brazil, but it is considered a *species inquirenda* as the holotype specimen has been lost and so its morphological characteristics and grouping cannot be confirmed (Pinto-da-Rocha et al. 2002). Species of this group also differ from the other western species by being larger, presenting chelicera with a higher number of teeth and basitibia of leg IV with four pseudoarticles (Giupponi and Miranda 2016).

Among the species from southeast Brazil, another common character is found, a sexual dimorphism in the pedipalps (Table 1, Figs 18, 19). With exception of the troglobite species *Charinus eleonorae* (which is distributed further north), males of *Charinus taboa* and all other *Charinus* species that occur in this region of the country (*Charinus montanus*, *Charinus brasilianus*, *Charinus asturius* and *Charinus jibaossu*) have longer pedipalps than the females (Pinto-da-Rocha et al. 2002, Vasconcelos et al. 2014, Weygoldt 1972). The presence of this characteristic among these species may indicate that they share the same recent common ancestor. Or, alternatively, organisms of these species might have undergone similar selective pressures in the past, which could have led to homoplasy in this character. Therefore, there is pressing need for a phylogenetic analysis of Brazilian *Charinus* species to understand these questions of relatedness between species.

**Table 1.** Measurements (mm) of body parts of the specimens of *Charinus taboa* sp. n.

		Males (n = 5)	Females (n = 5)
Total length		8.74 (6.56–11.12)	9.14 (6.02–10.85)
Cephalotorax	Length	3.31 (2.64–4.35)	3.21 (2.55–3.51)
	Width	4.69 (3.80–5.82)	4.63 (3.33–5.24)
Pedipalp	Femur	4.56 (2.23–9.26)	3.11 (2.01–3.60)
	Patella	5.02 (2.62–10.19)	3.30 (2.14–3.92)
	Tibia	1.43 (0.98–2.06)	1.34 (0.80–1.56)
	Tarsus	1.03 (0.78–1.42)	0.96 (0.66–1.08)
	Claw	0.72 (0.68–0.94)	0.74 (0.44-0.86)

*Charinus taboa* differs from *Charinus montanus*, *Charinus brasilianus*, *Charinus asturius* and *Charinus jibaossu* by having less developed eyes, and with the exception of *Charinus brasilianus*, five thricobotria instead of six in each series of the basitibia of leg IV (Fig. 17). *Charinus taboa* also differs from *Charinus montanus* in the shape of the segments of the sternum. While the second and third segments of the sternum of *Charinus taboa* are rounded (Figs 8, 11), in *Charinus montanus* these segments are flattened. *Charinus taboa* differs from *Charinus jibaossu* by having four spines in the ventral side of the patella (Fig. 6) instead of three. The quantity of spines on the others segments of the pedipalps is similar among many of the cited species, which makes it difficult to separate them by this characteristic alone (Weygoldt 1972, Pinto-da-Rocha et al. 2002, Vasconcelos et al. 2014).

*Charinus taboa* differs from *Charinus acaraje*, *Charinus troglobius*, *Charinus eleonorae*, *Charinus potiguar*, *Charinus caatingae* and *Charinus iuiu* mainly by the fact that these species have shorter pedipalps, with fewer amount of spines on the femur and patella, and from *Charinus mysticus* and *Charinus caatingae* by the presence of three spines on the tarsus of the pedipalps. *Charinus mysticus*, *Charinus acaraje*, *Charinus eleonorae*, *Charinus potiguar*, *Charinus caatingae* and *Charinus iuiu* also have the frontal and caudal series of the leg IV with six thricobotria each, *Charinus eleonorae* has a pointed frontal process, and in *Charinus troglobius*, the tritosternum is lacking the typical cone shape (Baptista and Giupponi 2002, 2003, Giupponi and Kury 2002, Pinto-da-Rocha et al. 2002, Vasconcelos et al. 2013, Vasconcelos and Ferreira 2016).

The morphologies of the male gonopod are quite variable among *Charinus* species; however, in dorsal view the shapes of the genital organ and LoD of *Charinus taboa* (Fig. 15) are similar to those in *Charinus eleonorae*. The female gonopod of *Charinus taboa* (Fig. 14) has the width larger than its length, which makes it similar to those of *Charinus asturius*, *Charinus eleonorae* e *Charinus mysticus*. Besides that, *Charinus taboa* presents its gonopod with the edges similar to that of *Charinus mysticus*, yet its shape is more irregular (Pinto-da-Rocha et al. 2002, Giupponi and Kury 2002, Baptista and Giupponi 2003).

The newly described species presents poorly developed eyes, lighter coloration than other non-troglobite species of *Charinus*, and is, to our knowledge, restricted to only two caves, which make plausible its status of troglobitic. Different degrees of troglomorphisms may appear due to changes in environmental conditions and not necessarily depends on cave occupancy by the organism. According to Weygoldt and Van Damme (2004), if a given region goes through changes in climate and consequently in humidity, organisms adapted to the anterior conditions can retreat in caves, which may result in troglomorphisms after some time, such as reduction in eye size and coloration, and elongation of legs and other appendages. Changes engendered by the restricted life in the interior of subterranean habitats in *Charinus* are the reduction in eye size, being completely absent in some cases, lightening of the color of the cuticle, change in the position of the pedipalps, being vertical in relation to the body, and elongation of the spines on the pedipalps (Baptista and Giupponi 2002, Baptista and Giupponi 2003, Weygoldt and Van Damme 2004, Delle Cave et al. 2009).

There are few troglobitic species of *Charinus* around the world: four in Brazil (*Charinus troglobius*, *Charinus eleonorae*, *Charinus caatingae*, *Charinus ferreus*), two in Venezuela (*Charinus tronchonii* (Ravelo, 1975) and *Charinus bordoni* (Ravelo, 1977)), and three in the Arabian Peninsula (*Charinus socotranus* Weygoldt, Pohl and Polak, 2002, *Charinus stygochthobius* Weygoldt and Van Damme, 2004, *Charinus omanensis* Delle Cave, Gardner and Weygoldt, 2009). *Charinus stygochthobius* represents the most troglomorphic species of those already described, since it lacks all its eyes, its cuticle is almost transparent, it has the pedipalps forming an angle of 45° in relation to the body and long spines on the pedipalps (Weygoldt and Van Damme 2004). *Charinus troglobius* presents total absence of the median eyes and small eyespots replacing the lateral eyes, depigmentation of the body, rotation of the pedipalps, as occurs to *Charinus stygochthobius*, and elongated spines (Baptista and Giupponi 2002). *Charinus eleonorae* has reduced lateral eyes, eyespots in the place of median eyes, light coloration, rotation of the pedipalps and elongated spines (Baptista and Giupponi 2003). *Charinus caatingae* presents lateral eyes reduced to eyespots, light pigmentation, and rotation of pedipalps in some individuals, being the least troglomorphic species in the country. *Charinus ferreus* presents almost complete loss of eyes and little coloration of the cuticle.

In the case of *Charinus taboa*, the eyes are still present, but they are smaller than those of most *Charinus* species in Brazil. In addition, this species has lighter coloration of the body compared to other species. However, some specimens of *Charinus taboa* present pigmented lateral eyes with a lesser degree of reduction (Figs 2, 9). The varying degree of troglomorphism within a population is common, although not well studied, and may be a characteristic of various groups. One example is the isopod *Asellus aquaticus*, which has polymorphisms in the degree of pigmentation of the eyes and body, in the size of the sensory appendages and body proportions (Prevorcnik et al. 2004).

With a cladistic analysis unavailable, it is not possible to ascertain whether a species of *Charinus* is troglobitic based solely on troglomorphic characters, since the species may have not been in a cave for sufficient time to develop morphological adaptations (beyond other factors, as the original size of the isolated population, species variability, etc.). Therefore, the condition of a given species of maintaining a viable population strictly inside caves should also be taken into account when deciding on the classification of a cave-dwelling species (Bolfarini and Bichuette 2015). This might be the case of *Charinus taboa*, which presents few troglomorphic characteristics, but is found within two caves located in a heavily deforested and human-modified landscape; the species seems to be unable to establish populations outside of its existing range. Yet, despite *Charinus taboa* has morphological characteristics suggesting the exclusive subterranean habit, more inventories are needed to confirm the non-occurrence of the species outside of these habitats.

Many species of *Charinus* in Brazil are highly vulnerable to extinction as a result of vast destruction of their habitat by deforestation or mining. *Charinus taboa*, which was recorded in only two very close caves, is considered rare and endemic. Thus, according to the laws of Brazil, this species may increase the biological importance of both the Taboa and BR-24 caves, and therefore ensures the continued preservation of those unique habitats.

## Supplementary Material

XML Treatment for Charinus

XML Treatment for Charinus taboa
